# Comprehensive Assessment of Host Responses to 5-Fluorouracil-Induced Oral Mucositis through Transcriptomic Analysis

**DOI:** 10.1371/journal.pone.0135102

**Published:** 2015-08-12

**Authors:** Chung-Ta Chang, Chien-Yun Hsiang, Tin-Yun Ho, Ching-Zong Wu, Hsiang-Hsi Hong, Yi-Fang Huang

**Affiliations:** 1 Department of Emergency Medicine, Far Eastern Memorial Hospital, Taipei, 22056, Taiwan; 2 School of Dentistry, College of Oral Medicine, Taipei Medical University, Taipei, 11031, Taiwan; 3 Department of Microbiology, China Medical University, Taichung, 40402, Taiwan; 4 Graduate Institute of Chinese Medicine, China Medical University, Taichung, 40402, Taiwan; 5 Department of Dentistry, Taipei Medical University Hospital, Taipei, 11031, Taiwan; 6 Department of Dentistry, Lotung Poh-Ai Hospital, Yilan, 26546, Taiwan; 7 Department of Periodontics, Chang Gung Memorial Hospital, Linkou, 33305, Taiwan; 8 Graduate Institute of Dental and Craniofacial Science, Chang-Gung University, Taoyuan, 33302, Taiwan; 9 Department of General Dentistry, Chang Gung Memorial Hospital, Linkou, 33305, Taiwan; Public Health Research Institute at RBHS, UNITED STATES

## Abstract

**Background:**

Chemotherapy plays an important role in current cancer therapy; however, several problems remain unsolved on the issue of host-therapeutics interaction. The purpose of this study was to investigate the host responses after 5-flurouracil (5-FU) administration and to find the target genes and their relationship with other cytokines in the 5-FU-induced oral mucositis (OM) mouse model through transcriptomic analysis.

**Materials and Methods:**

Thirty-six 6 to 8 week-old male BALB/c mice were randomly divided into the control group and 5-FU-treated group. In the 5-FU group, mice received 5-FU (100 mg/kg, intraperitoneally) on day 1, day 8, day 15, day 22, and day 29, respectively. We evaluated the oral mucosal change under macroanalysis and histological examination at indicated periods, and then applied transcriptomic analysis of gene expression profile and Immunohistochemical stain to identify the target molecules related to 5-FU-induced OM.

**Results:**

The most prominent histological change in this model was observed in the fifth week. The gene expression of Bone gamma-carboxyglutamate protein, related sequence 1 (Bglap-rs1) (–12.69-fold) and Chitinase 3-like 4 (Chi3l4) (–6.35-fold) were significantly down-regulated in this phase. The quantitative real-time PCR results also revealed the expression levels were 0.62-fold in Bglap-rs1 and 0.13-fold in Chi3l4 compared with the control group. Immunohistochemical stain showed significant expression of cluster of differentiation 11b (*p*<0.01), interleukin-1β (*p*<0.001) and tumor necrosis factor-α (*p*<0.05), and down-regulation of Bglap-rs1 (*p*<0.01) compared with the control group. By Kyoto Encyclopedia of Genes and Genomes pathway analysis, there were twenty-three pathways significantly participated in this study (*p*<0.05).

**Conclusions:**

Through comprehensively transcriptomic analysis and IHC stain, we discovered several valuable pathways, verified the main pro-inflammatory cytokines, and revealed two significantly down-regulated genes in the 5-FU-induced OM model. These findings highlighted the way of seeking effective therapeutic agents for chemotherapy-induced OM in future.

## Introduction

5-Fluorouracil (5-FU) is a popular and effective chemotherapy drug that is recommended in the treatment of many types of cancers, including colon cancer, breast cancer, and head and neck cancers [1]. However, many adverse effects, such as mucositis, myelosuppression, headaches, dermatitis, photophobia, diarrhea, and cardiotoxicity can occur during the therapeutic period [1,2]. About 20% to 40% of patients experience oral mucositis (OM) when receiving conventional chemotherapy, and a high-dose protocol can even produce severe OM rates in approximately 80% of patients [3]. OM is a painful lesion characterized by erythema or ulceration of the oral mucosa, and local or systemic infections will sequentially develop on the injured mucosal barrier [4]. This disease is not only associated with symptoms of discomfort and the lowering of patients’ life quality, but also significantly increases hospital costs as well as prohibiting cancer treatment [5–7].

Historically, chemotherapy-induced OM was believed to be an epithelium-mediated event, which represented the consequence of the nonspecific harmful effects on stem cell differentiation [8]. The renewal capacity of the epithelium will be deprived after this kind of direct damage to the basal epithelial cell layer by chemotherapy drugs, resulting in clonogenic cell death, mucosal atrophy, and consequent ulceration [9]. Although direct cell injury is the critical first step to initiate the process of OM, the pathological mechanism of cytotoxic agents related to OM is very complex. Several authors have suggested that reactive oxygen species generated by cyclooxygenase-2 and inducible nitric oxide synthase in injured cells leads to the development of OM [10,11]. Subsequently, the expression of pro-inflammatory cytokines, such as tumor necrosis factor-α (TNF-α) and interleukin-1 (IL-1), are increased significantly [9,12]. Logan et al. [11] stated the expression of nuclear factor-kappaB was increased significantly according to the post-chemotherapy immunohistochemistry (IHC) stain of biopsies from twenty chemotherapy-induced OM patients. Moreover, Sonis [9] concluded TNF-α could give a positive feedback to NF-κB and amplify its response, and as a result mitogen-activated protein kinase signaling could be activated. Due to the development of genetic screening tools in the past decade, genes involving in the chemotherapy-induced OM have also been delineated [13].

In spite of the fact that the microarray has become a popular investigation tool in the research of chemotherapy-induced OM [14,15], the pivotal molecules and the relationship between these activated genes are still not clearly understood, and improved treatment compounds that target at the pivotal molecules of OM remain to be developed. The purpose of this study was to investigate the host responses after 5-FU administration, with the aim to find the target genes and their relationship with other cytokines in the 5-FU-induced OM mouse model through transcriptomic analysis. We investigated the fundamental impacts of the 5-FU-affected transcriptomic pattern and expect that our findings would be helpful for future cancer therapy research.

## Materials and Methods

### Animal experiments

Mouse experiments were controlled under the ethics approval from China Medical University Animal Ethics Committee (Permit Number: 97-28-N) and the study was performed in accordance with the guidelines laid down by the National Institute of Health in the USA regarding the care and use of animals for experimental procedures. BALB/c mice were obtained from National Laboratory Animal Center (Taipei, Taiwan).

The 5-FU-induced OM mouse animal model was modified according to a previous 5-FU-induced OM study [16]. Thirty-six 6 to 8 week-old male mice were randomly divided into two groups, including 18 mice for the control group and 18 mice for the 5-FU group, and they were administered intraperitoneally (i.p.) with phosphate-buffered saline (PBS) (137 mM NaCl, 1.4 mM KH_2_PO_4_, 4.3 mM Na_2_HPO_4_, 2.7 mM KCl, pH 7.2) or 5-FU (100 mg/kg) (Sigma, St. Louis, MO, USA) sequentially on day 1, day 8, day 15, day 22, and day 29, respectively. No mechanical trauma to oral cavity was performed on any of the mice. Then the Mice were humanely killed with 2% thiopental (80 mg/kg, i.p.) for histological examination on day 3 (the 1^st^ week), day 17 (the 3^rd^ week), day 31 (the 5^th^ week), and day 45 (the 7^th^ week). All animals were housed in an animal room and had access to pelleted food and water *ad libitum* under standard laboratory conditions in a temperature-controlled room with a 12-hour light/dark cycle. No mice in the control group died, but one mouse in the 5-FU group died during the experimental period.

### Macroanalysis and histological examination

Mice were subjected to blinded macroscopic oral mucosal assessment on day 3, day 10, day 17, day 31, and day 45. Macroscopic assessment of the OM severity was scored based on a previous study [10], and the average points were calculated and recorded. The criteria we used to investigate the macroscopic damage and the numerical rating score were as follows: 0, normal oral cavity; 1, presence of erythema and hyperemia; 2, presence of hemorrhagic areas or small ulcerations; and 3, extensive ulcerations or abscesses.

In histological examination, at least three of the 5-FU-treated mice were sacrificed in every indicated period. After fixing the oral mucosal samples in 10% (v/v) phosphate-buffered formalin solution for 2 days, the samples were rinsed in saline, and sequentially dehydrated in a series of graded alcohols (50% (v/v), 70% (v/v), and 95% (v/v)) for 30 minutes each. They were then embedded in paraffin, cut into 5-μm sections, stained with hematoxylin and eosin (H&E), and subjected to blinded histological assessment. Histological changes were graded semi-quantitatively from 0 to 4 which were adapted from a previous study [10]. Briefly, the criteria we used to investigate the microscopic change were as follows: 0, normal epithelium and connective tissue; 1, discreet vasodilatation and inflammatory infiltration with mononuclear prevalence; 2, moderate vasodilatation and inflammatory infiltration with neutrophil prevalence, and the presence of hemorrhagic areas, and edema; 3, severe vasodilatation and inflammatory infiltration with neutrophil prevalence, and the presence of hemorrhagic areas, edema and ulceration but no abscesses; and 4, severe vasodilatation and inflammatory infiltration with neutrophil prevalence, and the presence of hemorrhagic areas, edema and extensive ulceration and abscesses.

### Microarray analysis

Total RNA was extracted from each mouse’s oral mucosa using RNeasy Mini kit (Qiagen, Valencia, CA, USA), and the total RNA was then quantified and evaluated. Microarray analysis was executed as previously described [17,18]. Briefly, after the fluorescent-labeled, RNA targets were produced from 5 μg of total RNA by MessageAmp aRNA kit (Ambion, Austin, TX, USA) and Cy5 dye (Amersham Pharmacia, Piscataway, NJ, USA), they were hybridized to the Mouse Whole Genome OneArray (Phalanx Biotech Group, Hsinchu, Taiwan), and an Axon 4000 scanner (Molecular Devices, Sunnyvale, CA, USA) was applied to scan all of the samples. At least three replicates were accomplished from each mouse. The Genepix 4.1 software (Molecular Devices) was applied to examine the Cy5 fluorescent intensity of each spot, and the spots that had a signal-to-noise ratio of less than 0 or the control probes would be filtered out. The others that met the requirements of these criteria were normalized by R program [19]. The normalized signal intensities of genes in 5-FU-treated mice were divided with those in PBS-treated mice to calculate the fold changes of genes. The Transcription Regulation algorithm in the MetaCore Analytical suite (GeneGo Inc., St. Joseph, MI, USA) was used to generate the biological network which input genes were selected as fold changes ≥2 or ≤-2.

### Quantitative real-time PCR (qPCR)

The expression levels of Chitinase 3-like 4 (Chi3l4) and Bone gamma-carboxyglutamate protein, related sequence 1 (Bglap-rs1) genes were validated by qPCR. RNA samples were transcribed reversely for 2 hours at 37°C with the High Capacity cDNA Reverse Transcription Kit (Applied Biosystems, Foster City, CA, USA) and the qPCR was executed by using 1 μl of cDNA, 2× SYBR Green PCR Master Mix (Applied Biosystems), with 200 nM of forward and reverse primers. The reaction condition was followed: 10 minutes at 95°C; 40 cycles of 95°C for 15 seconds; 1 minute at 60°C. Each assay was run on an Applied Biosystems 7300 Real-Time PCR system in triplicates. Fold changes were calculated using the comparative C_T_ method. The primer sets for each gene were as follows: Bglap-rs1 forward, 5′-GACCCTCTCTCTGCTCACTC-3′; Bglap-rs1 reverse, 5′-TCACTACCTTATTGCCCTCCTG-3′; Chi3l4 forward, 5′-CCTAAGAATGGCTACACTGGAG-3′; Chi3l4 reverse, 5′-TGCTGGAAATCCCACAATGAG-3′; glyceraldehyde-3-phosphate dehydrogenase (GAPDH) forward, 5′-ACACCCACTCCTCCACCTTT-3′; GAPDH reverse, 5′-TAGCCAAATTCGTTGTCATACC-3′.

### Immunohistochemical staining

Mice were treated with 5-FU and their oral mucosa was collected 5 weeks later for IHC staining. Briefly, parafilm-embedded oral mucosa was cut into 5-μm sections, incubated with anti-cluster of differentiation 11b (anti-CD11b), anti-IL-1β, anti-TNF-α, or anti-Bglap-rs1 antibodies (Santa Cruz, Dallas, TX, USA) overnight at 4°C, and then incubated with biotinylated secondary antibodies (Zymed Laboratories, Carlsbad, CA, USA). Finally, sections were incubated with avidin-biotin complex and stained with 3,3'-diaminobenzidine (Histostain-Plus, Zymed Laboratories, Carlsbad, CA, USA). The proportions of positive area (%) were calculated using ImageJ (http://imagej.nih.gov/ij/index.html).

### Statistical analysis

Data were presented as mean ± standard error. The Student’s *t*-test was used to find comparisons between the two experiments. A value of *p*<0.05 was considered statistically significant.

## Results

### Macroanalysis and histological examination of the oral mucosa following administration of 5-FU

**Fig 1:**
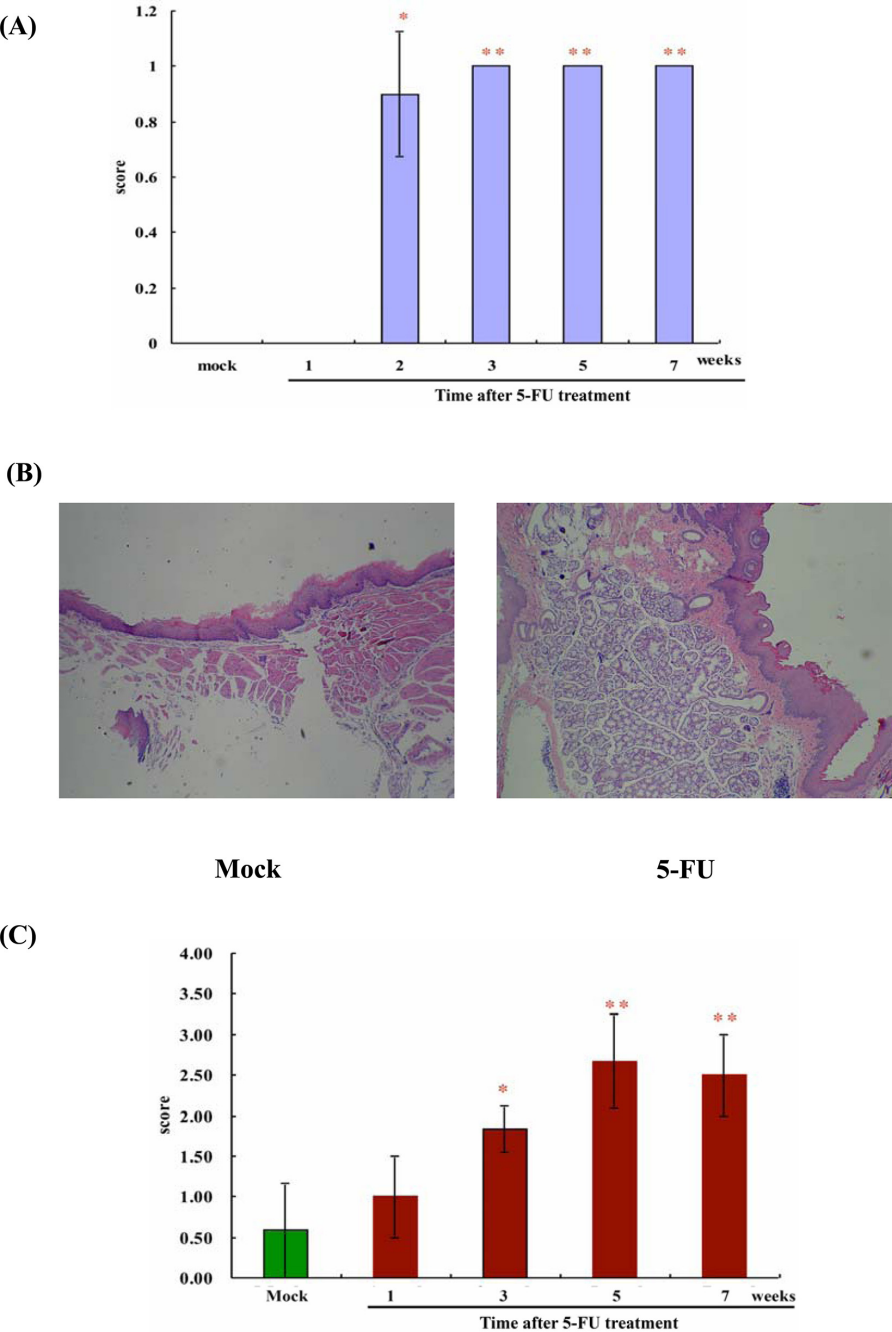
Macroscopic and microscopic examinations of the oral mucosa following 5-FU administration. BALB/c mice were intraperitoneally administered with PBS (mock, the control group) or 5-FU and investigated at indicated periods. (A) Macroanalysis. Oral mucositis was induced via intraperitoneal administration of 5-FU. The oral cavity of each mouse was evaluated. Erythema and hyperemia of oral mucosa were significantly observed in the second week after 5-FU administration (*p*<0.05). During the third to seventh week period, the significant difference between 5-FU-induced oral mucositis and the control group (mock) persisted (*p*<0.01). (B) Microscopic features of the oral mucosa. In the fifth week, three mock mice and four 5-FU-treated mice were sacrificed and the sections were stained with H&E and observed using light microscope. Magnification 100×. Photos are representative images. Histopathologically, the oral mucosa of mice subjected to 5-FU-induced oral mucositis showed accentuated vasodilatation, intense cellular infiltration with neutrophil prevalence, hemorrhagic areas, edema, and extensive ulcers, compared with the control group (mock). (C) According to the oral mucositis microscopic analysis scoring system, a significant difference was noted in the third week for the first time (*p*<0.05), and the peak influence was noted in the fifth week (*p*<0.01). Then the inflammatory condition was relieved in the seventh week investigation (red column: 5-FU treatment; green column: mock, the control group). Values are mean ± standard error. *p<0.05, **p<0.01, compared with the control group (mock).

When compared with the control group, on the oral mucosa of mice intraperitoneally administrated with 5-FU we found significant lesions (*p*<0.05) in macroanalysis, which were represented mostly by prominent erythema. Significant clinical signs were found two weeks after 5-FU administration (*p*<0.05) (Fig 1A). However, no lesion presented hemorrhages, extensive ulcers, and abscesses in macroanalysis during the experimental period.

In the fifth week, histological examination of the oral mucosa of mice administrated with 5-FU showed remarkable vasodilatation, intense inflammation with neutrophil prevalence, hemorrhagic areas, edema, and extensive ulcers, when compared with the control group (Fig 1B). These findings indicated that the intraperitoneal injection of 5-FU did induce mucosal damage and subsequent tissue inflammation. From the histological examination and scoring system, we observed that the inflammatory process had become significant since the third week onwards (*p*<0.05) and the severity of OM persisted until the seventh week during the experimental period (Fig 1C).

### Transcriptomic analysis of oral mucosa after 5-FU was administered

Microarray data were computed by the Gene Expression Pattern Analysis Suite v3.1 to test for the gene expressed with fold changes >2.0 or ≤–2.0 in the mouse oral mucosa with 5-FU-induced OM. In a total of 30,968 genes, the transcripts of 19 genes were significant expressed in the 5-FU-treated group (Table 1). The microarray data were then analyzed by the Parametric Gene Set Enrichment Analysis (PGSEA) package of a bioconductor program to get enriched gene sets for the mice of 5-FU-induced OM, and we found Bglap-rs1 and Chi3l4 were significantly down-regulated (–12.69-fold and −6.35-fold when compared with the control group, respectively). Table 1 listed the gene sets in lesion with 5-FU-induced OM of mice.

**Table 1:** The expression levels of significantly changed genes induced by 5-FU in oral mucositis.

Accession	Gene Symbol	Gene Description	Fold changes
NM_031368.4	Bglap-rs1	Bone gamma-carboxyglutamate protein, related sequence 1	-12.69
NM_145126	Chi3l4	Chitinase 3-like 4	-6.35
NM_011468	Sprr2a	Small proline-rich protein 2A	-2.66
NM_026576	Etaa1	Ewing's tumor-associated antigen 1 homolog.	-2.59
NM_207547	V1rd21	Vomeronasal 1 receptor, D21	-2.48
NM_026046	Zfp329	Zinc finger protein 329	-2.27
NM_008648	Mup4	Major urinary protein 1	-2.17
NM_133239	Crb1	Crumbs homolog 1 (Drosophila)	-2.01
NM_010733	Lrrn3	Leucine rich repeat protein 3, neuronal	2.02
NM_031255	Rshl1	Radial spokehead-like 1	2.09
NM_023371	Pin1	Protein (peptidyl-prolyl cis/trans isomerase) NIMA-interacting 1	2.11
NM_026334	Lipf	Lipase, gastric	2.11
NM_007832	Dck	Deoxycytidine kinase	2.14
NM_009499	Vasp	Vasodilator-stimulated phosphoprotein	2.35
NM_021435	Slc35b4	Solute carrier family 35, member B4	2.43
NM_026132	Txndc8	Thioredoxin domain containing 8	2.58
NM_030749	Sil1	Nucleotide exchange factor SIL1 precursor.	2.62
NM_178408	Arrdc1	Arrestin domain containing 1	2.85
NM_008190	Guca2a	Guanylate cyclase activator 2a (guanylin)	11.55

Through the Kyoto Encyclopedia of Genes and Genomes (KEGG) pathway analysis, we learned that twenty-three pathways were significantly involved in the 5-FU-induced OM model (Table 2). For example, the *epidermal growth factor* (EGF) signaling pathway (*p* = 0.0007233), the granulocyte colony-stimulating factor (G-CSF) signaling pathway (*p* = 0.016483), and the transforming growth factor beta 1 (TGF-β1) signaling pathway (*p* = 0.038104) were related with epithelial healing, blood cell maturation, and epithelial cell mitosis inhibition, respectively; more details are shown in Table 2.

**Table 2:** KEGG pathway analysis of genes in oral mucosa at the 5^th^ week after intraperitoneally administered with 5-FU.

KEGG pathway^a^	*p* value^b^
EGF Signaling Pathway	0.007233
Arginine and proline metabolism	0.007258
Regulation of actin cytoskeleton	0.008047
IFN-β Signaling Pathway	0.009013
Citrate cycle (TCA cycle)	0.01004
Glyoxylate and dicarboxylate metabolism	0.013511
Nef Mediated Pathway	0.013886
Steroid Hormone Signaling Pathway	0.016138
G-CSF Signaling Pathway	0.016483
One carbon pool by folate	0.021077
Farnesyltransferase Signaling Pathway	0.023384
Antigen processing and presentation	0.023434
Cell Communication	0.028426
Angiotensin Signaling Pathway	0.031787
CCR2 Mediated Pathway	0.033148
Limonene and pinene degradation	0.033356
B Cell Antigen Receptor Signaling Pathway	0.034871
EPO Signaling Pathway	0.03591
TGF-β1 Signaling Pathway	0.038104
PRL Signaling Pathway	0.039095
Leukotriene Signaling Pathway	0.039145
DHT Signaling Pathway	0.040799
Urea cycle and metabolism of amino groups	0.046441

^a^ Genes with fold changes >2.0 or ≤-2.0 were analyzed by KEGG pathways

^b^ *p* values were calculated by the geneSetTest function implemented in the limma package

### Verification of the target genes by qPCR

According to qPCR analysis, we discovered that Bglap-rs1 and Chi3l4 had significantly different gene expression in the 5-FU-treated group. To quantify these two gene expressions, we calculated that the chemotherapy drug 5-FU down-regulated the Bglap-rs1 and Chi3l4 gene expressions in 0.62- and 0.13-fold respectively, and both of them were significantly down-regulated in the 5-FU-treated group compared with the control group (*p*<0.01) (Table 3).

**Table 3:** The expression levels of Bglap-rs1 and Chi3l4 genes by qPCR.

Sample	Average C_T_ of target	Average C_T_ of GAPDH	ΔC_T_ ^a^	ΔΔC_T_ ^b^	Relative to Mock
Bglap-rs1					
Mock	22.81±0.09	18.80±0.03	4.00±0.09	0.00±0.09	1.00
5-FU administered	22.95±0.02	18.26±0.04	4.68±0.05	0.67±0.05	0.62**
Chi314					
Mock	18.91±0.07	18.80±0.03	0.10±0.07	0.00±0.07	1.00
5-FU administered	21.21±0.08	18.26±0.04	2.95±0.10	2.84±0.10	0.13**

^a^ The ΔC_T_ value is determined by subtracting the average GAPDH C_T_ value from the average target gene C_T_ value. The standard deviation of the difference is calculated from the standard deviations of the target gene and GAPDH

^b^ The calculation of ΔΔC_T_ involves subtraction by the ΔC_T_ calibrator value. This is a subtraction of an arbitrary constant, so the standard deviation of ΔΔC_T_ is the same as the standard deviation of the ΔC_T_ value

**p<0.01, compared with the control group (mock)

### Immunohistochemical staining analysis of oral mucosa following 5-FU administration

**Fig 2:**
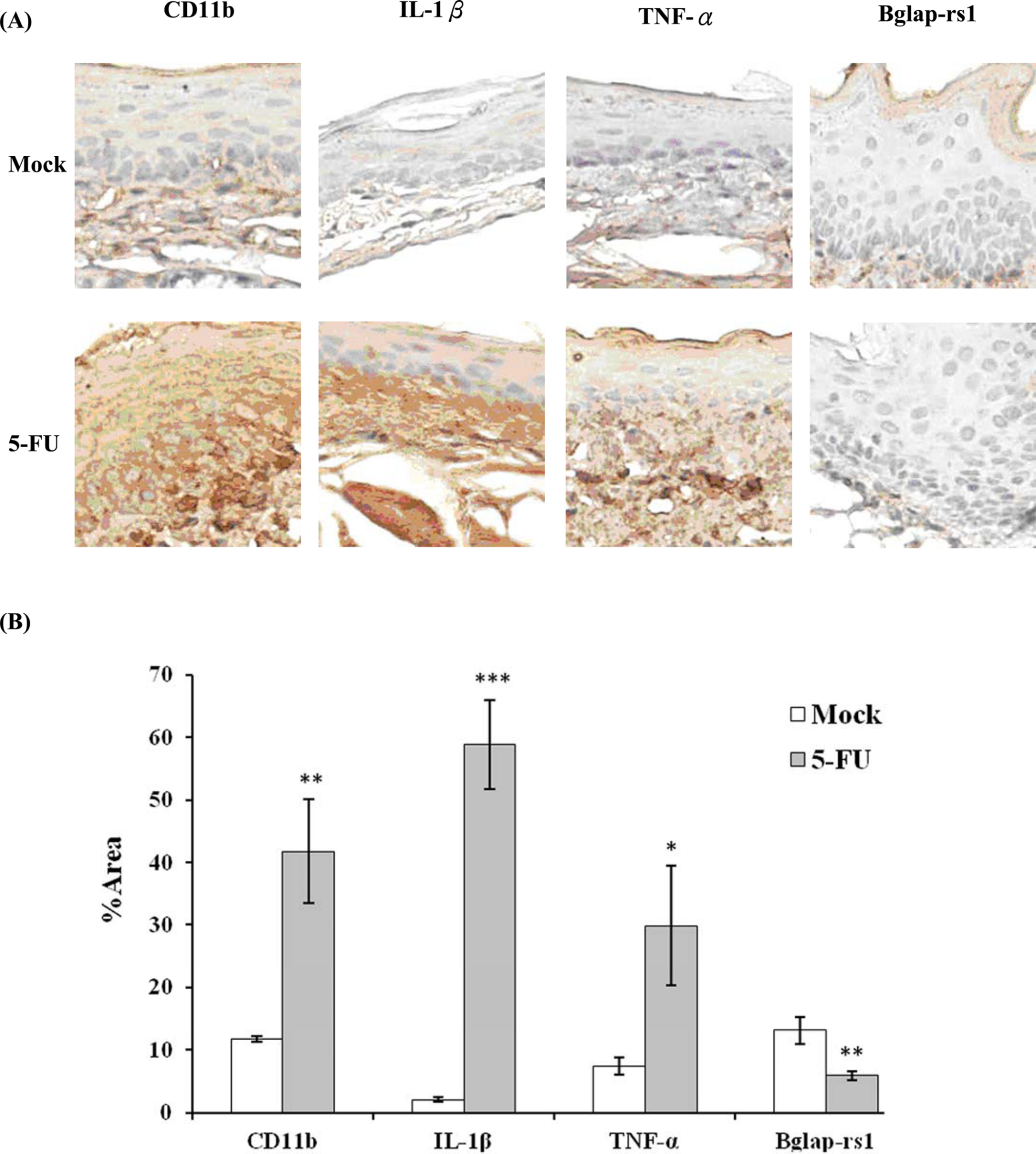
Immunohistochemical staining of 5-FU-treated oral mucosa. Mice were treated with 5-FU and oral mucosa was collected 5 weeks later. Sections of oral mucosa were stained with antibodies against CD11b, IL-1β, TNF-α, or Bglap-rs1. (A) Immunohistochemical staining (200× magnification). Photos are representative images (n = 5 /group). (B) Quantitation of photos. Results are expressed as area (%). Values are mean ± standard error (n = 5 /group). *p<0.05, **p<0.01, ***p<0.001, compared with the control group (mock).

IHC staining was performed to verify 5-FU-induced inflammation in oral mucosa. CD11b is the marker of monocytes and granulocytes. The increase of CD11b-positive area in oral mucosa after 5-FU treatment (*p*<0.01) suggested the infiltration of inflammatory cells (Fig 2). Moreover, the production of pro-inflammatory cytokines, such as IL-1β and TNF-α, was increased (*p*<0.001 and *p*<0.05, respectively), which suggests that 5-FU-induced inflammation in oral mucosa. In addition, the Bglap-rs1-positive area decreased in oral mucosa (*p*<0.01), confirming that 5-FU down-regulated the expression of the Bglap-rs1 gene in 5-FU treated oral mucosa.

## Discussion

5-FU is a chemotherapy medicine frequently used for the treatment of colon, breast, and head and neck malignancies. It eliminates cancerous cells through impeding DNA and protein synthesis [1]. However, OM and diarrhea are common side effects, which occur in about 80% of patients receiving 5-FU therapy. In this study, the pathological change of 5-FU-induced OM was investigated at indicated periods by both macroanalysis and histological examination; thus, the specimens could be obtained according to the severity of 5-FU-induced OM for further transcriptomic analysis. During the experimental period, the severity of OM progressed with increasing time. In the histological examination, the oral mucosa of 5-FU-treated group showed remarkable vasodilatation, intense inflammation with neutrophil prevalence, hemorrhagic areas, edema, and extensive ulcers in the fifth week. Additionally in the IHC stain, CD11b, a marker of monocytes and granulocytes, and two important pro-inflammatory cytokines, IL-1β and TNF-α, were all significantly up-regulated in this phase. These findings are in agreement with previous studies [9,20] and therefore we suggest that 5-FU is a potent chemotherapy agent in inducing OM [21]. Although OM change was significantly observed in the second week according to macroanalysis, this result was compatible with a previous study with human subjects [8]. However, no hemorrhages, extensive ulcers, or abscesses were found in macroanalysis, which we suggest might be due to the smaller oral cavities and lack of cheek pouches in mice.

Many researches used DNA microarray to investigate gene expression patterns and forecasted the clinical outcome and prognosis of patients receiving chemotherapy [22,23]. In our previous study, we used the microarray tool to identify that the nuclear factor-κB may be the pivot molecule in the 5-FU-induced intestinal mucositis development [24]. In this study, we further investigated the cell response after 5-FU stimulation, and found that Chi3l4 and Bglap-rs1 are significantly down-regulated in the mouse animal model of 5-FU-induced OM.

According to KEGG pathway analysis of the genes involved in the 5-FU-induced OM model, we learned that OM is a very complex inflammatory process and discovered twenty-three pathways that participated significantly. For example, EGF signaling pathway controls many cellular processes including epithelial cell proliferation, migration, and survival [25]. Several reports concluded decreased salivary EGF is related with more severe OM [26,27], and found EGF plays a role in accelerated wound healing and tissue regeneration [28]. G-CSF not only plays an important role in the maturation of bone marrow progenitor cells to form neutrophils, basophils, and eosinophils, but also contributes to the stimulation of fibroblast and epithelial cell regeneration by augmenting IL-1 transcription and translation in OM [29]. Consequently, G-CSF [30] and IL-1 [31] were suggested to have roles in prevention and treatment of OM. Moreover, CD11b was suggested to be a marker of activation of neutrophils and macrophages, and the quantity of CD11b in neutrophils represented their activation in the inflammatory process [32]. Several reports also demonstrated G-CSF is a potent stimulator for the expression of CD11b on the surface of neutrophils [33,34]. In this study, both CD11b and IL-1β were significantly activated and neutrophil prevalence was discovered indicated the inflammatory property of 5-FU-induced OM. Therefore, we suggest that G-CSF could play an important role in 5-FU-induced OM. TGF-β1 is the main form of TGF-β in keratinocytes [35]; it is able to play as an antiproliferative factor in epithelial cells and endothelial cells. These cells will be arrested in the G1-phase, hence, TGF-β is supposed to have the possibility to reduce OM [36]. Taken together, these participated pathways provide researchers with a novel route to seek effective therapeutics for chemotherapy-induced OM. Many agents have been investigated to ameliorate the course of OM, such as Keratinocyte growth factors [37], EGF [38], G-CSF [39], and TGF-β [40]. In this study many valuable pathways were identified, and we hope these findings could highlight the treatment path of chemotherapy-induced OM in the future.

Chi3l4 is a kind of chitinase-like proteins (CLPs) in rodents, and it has been proposed that Chi3l4 participates in the processes of the carbohydrate metabolism, chitin catabolism, and inflammatory response [41]. Webb et al. [42] suggested IL-4 might increase the expression of Chi3l4 mRNA in a STAT6-dependent fashion in the mouse model of ovalbumin-induced allergic airway inflammation. The main producers of Chi3l4 are macrophages, dermal, mast cells, and dendritic cells, and Chi3l4 is determined to contribute to the process of fibrosis and tissue repair, thus resulting in remodeling of the extracellular matrix [43]. Additionally, Qureshi et al. [44] revealed overexpression of CLPs in the dermis of the nasopharyngeal carcinoma mouse model, and they stated that CLPs are critical therapeutic targets to limit inflammation in cancer. In this study, the Chi3l4 gene was significantly down-regulated after 5-FU administration; we suggest that 5-FU could be a potent CLPs inhibitor and it might have a chance to become a more effective drug in cancer therapy. Furthermore, since expression of Chi3l4 plays a role in tissue repair, we also suggest the down-regulation of Chi3l4 after 5-FU administration might contribute to the development of 5-FU-induced OM.

The human osteocalcin gene, Bglap, is secreted mainly by normal maturing osteoblasts and controls the dynamic process of bone resorption and new bone formation [45]. When Bglap is expressed, it also improves proliferation, adhesion, and the survival of tumor cells [46]. In contrast to Bglap, TNF-α plays an anti-tumor role, and it is able to inhibit the expression of Bglap [47]. Bglap is regarded as a TNF-α target and indirectly suggests a tumor-promoting function in cancers. There are three osteocalcin genes in mice, including osteocalcin gene 1, osteocalcin gene 2, and Bglap-rs1 [48]. In this study, TNF-α was significantly expressed in the IHC stain and Bglap-rs1 was significantly suppressed both in qPCR analysis and the IHC stain, hence we suggest there might be a connection between up-regulation of TNF-α and down-regulation of Bglap-rs1 in the 5-FU-induced OM mouse animal model. This condition might decelerate the process of oral mucosa regeneration and remodeling, and therefore OM could occur.

Since 5-FU-induced OM is a common medical problem, a number of approaches to manage the early oral mucosal changes by cytotoxic therapy have been proposed in current medical practices [49,50]. However, it is hard to find an effective pharmacologic or biologic agent for OM, because the cellular pathological mechanism is not yet clearly understood. In this study we found CD11b and two important pro-inflammatory cytokines, IL-1β and TNF-α, and twenty-three pathways that were significantly involved in the process of 5-FU-induced OM, and some of the growth factors have been proposed to ameliorate the severity of chemotherapy-induced OM, such as EGF, G-CSF, and TGF-β. Furthermore, we discovered two significantly down-regulated genes, Chi3l4 and Bglap-rs1, in the 5-FU-induced OM model, and suggest that they are relevant to the cytotoxic effect of 5-FU. According to previous studies, we suggest that these two genes might play a part in tissue homeostasis. The significant suppression of Chi3l4 and Bglap-rs1 might prohibit the process of tissue remodeling, as a consequence, ulceration in oral mucosa could easily form and the OM lesion could be more susceptible to bacterial infection in this situation.

## Conclusion

To our knowledge, this was the first report that comprehensively investigated the gene expression in 5-FU-induced OM mouse animal model. We demonstrated several valuable pathways, and some of the growth factors proposed have treatment potential according to previous studies. We also verified the important roles of CD11b, IL-1β and TNF-α in 5-FU-induced OM. In addition, two significantly down-regulated genes, Chi3l4 and Bglap-rs1, were discovered and we suggest that they might play a part in the process of 5-FU-induced OM.
